# Digital Patient Decision Aid for Antiobesity Medications: Mixed Methods Study of Human-Centered Design and Usability Evaluation

**DOI:** 10.2196/89428

**Published:** 2026-05-15

**Authors:** Li-Jen Wang, Yi-Jen Wang, Yu-Lun Cheng, Wen-Liang Fang, Weu Wang, Meng-Cong Zheng

**Affiliations:** 1 College of Design National Taipei University of Technology Taipei Taiwan; 2 Department of Family Medicine Taipei Veterans General Hospital Taipei Taiwan; 3 School of Medicine, College of Medicine National Yang Ming Chiao Tung University Taipei Taiwan; 4 Division of Cardiology Department of Internal Medicine Taipei Medical University Hospital Taipei Taiwan; 5 Department of Surgery Weight Management Medical Center Taipei Veterans General Hospital Taipei Taiwan; 6 School of Medicine College of Medicine National Yang Ming Chiao Tung University Taipei Taiwan; 7 Division of General Surgery Department of Surgery Taipei Medical University Hospital Taipei Taiwan; 8 Department of Surgery, School of Medicine College of Medicine Taipei Medical University Taipei Taiwan; 9 Department of Industrial Design National Taipei University of Technology Taipei Taiwan

**Keywords:** preference-sensitive decision-making, risk communication, shared decision-making, digital health interventions, obesity, Taiwan

## Abstract

**Background:**

The global burden of obesity continues to rise, highlighting the need for patient-centered approaches to weight management. Shared decision-making is particularly important in the selection of antiobesity medications (AOMs), as treatment options differ in mechanism, effectiveness, side effects, routes of administration, and cost. Despite this preference-sensitive context, only a few patient decision aids (PDAs) have been culturally and clinically adapted for use in Asian populations.

**Objective:**

This study aims to design, develop, and evaluate a digital PDA, OptiWeight, to support shared decision-making for AOM selection, incorporating perspectives from health care professionals and patients.

**Methods:**

This mixed methods, multicenter study, conducted between August 2022 and November 2025, applied a 4-stage human-centered design process. An evidence-informed prototype was developed based on clinical guidelines, followed by 2 rounds of usability testing using think-aloud protocols to assess navigation structures, perceived usability (System Usability Scale [SUS]), and cognitive workload (NASA Task Load Index [NASA-TLX]). Semistructured interviews with health care professionals specializing in weight management, guided by the Consolidated Framework for Implementation Research, informed clinical implementation and workflow integration. Finally, patients with overweight or obesity evaluated usability, cognitive workload, and overall user experience in outpatient settings. Qualitative data were analyzed using content analysis, and 1-way analysis of variance examined changes in usability and workload across stages.

**Results:**

A total of 174 individuals were included across all study stages (usability testing among adults: n=78; health care professional interviews: n=18; and clinical evaluation among patients: n=78). Iterative usability testing comparing system- and user-controlled navigation structures revealed complementary strengths and limitations, leading to the adoption of a hybrid navigation structure supporting both sequential guidance and flexible comparison. Additional design requirements included the use of icon arrays to enhance risk comprehension and localization features such as treatment cost displays and clarification of socially impactful side effects. Perceived usability increased from initial testing to clinical evaluation (SUS: 60.53-73.65, *P*<.001), meeting good usability thresholds, while cognitive workload decreased (NASA-TLX: 40.35-16.69, *P*<.001).

**Conclusions:**

Through a systematic human-centered design process integrating health care professional and patient perspectives, OptiWeight addresses the lack of culturally adapted PDAs for AOM decision-making in Mandarin-speaking populations while capturing user needs—particularly regarding navigation flexibility and risk visualization. The final tool demonstrated good usability and feasibility, and workflow considerations suggest potential for integration into routine weight-management care. Further research is needed to evaluate its impact on decision quality and real-world implementation outcomes.

## Introduction

The global burden of obesity continues to rise, with the World Obesity Atlas 2024 predicting that over half of the world’s population will be overweight or obese by 2035 [1]. In Taiwan, national survey data indicate that the combined prevalence of overweight and obesity among adults aged 18 years and older rose from 32.7% in 1993-1996 to 50.8% in 2022, exceeding half of the adult population [2,3]. These trends are not evenly distributed across population subgroups. Prevalence is consistently higher in men than in women (61.2% vs 41.5% in 2020-2024) and increases with age, with the highest rates observed among middle-aged adults (eg, 72.9% in men aged 35-44 years) [4]. In addition, approximately one-third of school-aged children are classified as overweight or obese, indicating that this trajectory extends across the life course [3]. Obesity is now widely recognized as a major risk factor for type 2 diabetes, cardiovascular disease, and numerous chronic conditions [5]. A range of evidence-based weight-management treatments is available, including lifestyle modification, medication therapy, and bariatric surgery [6]. Among these options, antiobesity medications (AOMs) have become increasingly common and effective in clinical practice [7]. As each medication differs in its mechanism of action, expected weight-loss outcomes, and potential side effects, there is no universally optimal choice. Treatment selection is therefore considered a preference-sensitive decision that should reflect the values and priorities of individual patients [8-10].

Shared decision-making (SDM) is particularly critical in such contexts. Clinicians are responsible for presenting the best available evidence, while patients—supported throughout the process—evaluate the options, clarify their preferences, and choose the alternative that best aligns with their values [11]. Patient decision aids (PDAs) are key tools that promote SDM by presenting clinical scenarios, summarizing the benefits and risks of available options, and helping patients articulate and communicate their values [12,13]. Randomized trials and systematic reviews have consistently demonstrated that PDAs increase patient knowledge, improve understanding of treatment trade-offs, reduce decisional conflict, and enhance patient-clinician communication [13,14]. These benefits are particularly meaningful for underserved populations—including those with lower health literacy, lower income, or limited access to specialist care—who often face greater challenges in understanding health information, participating in clinical decision-making, and achieving value-concordant decisions [15].

This is especially important in obesity care. In Taiwan, a volume-driven reimbursement system may limit communication and continuity of care [16], while short consultation times and limited provider training further constrain in-depth weight-management discussions [17]. At the patient level, unmet information needs may lead individuals to seek AOMs without professional guidance [18], and weight stigma may discourage engagement with formal health care [19], further exacerbating disparities in access to evidence-based care, particularly in settings where treatment costs are borne out of pocket, and specialized services are unevenly distributed [20].

Several PDAs have been developed in the obesity field, including decision aids for adolescent weight management [21] and tools supporting choices among bariatric surgery procedures [22-24]. These interventions have been shown to decrease decisional conflict and improve knowledge and satisfaction [21-24]. However, existing PDAs primarily target surgical decision-making [22-24] or specific subgroups such as adolescents and their parents [21], leaving a gap in tools designed for adults considering medication therapy. An environmental scan of existing weight-loss decision aids has further shown that many available tools do not meet key principles of the International Patient Decision Aid Standards (IPDAS), particularly regarding language localization and optimization of content for end users [25,26].

At the time of this study, no PDA in Taiwan specifically supported patients in choosing among AOMs, despite the clinical importance of such decisions. The recent introduction of several new AOMs, including glucagon-like peptide-1 receptor agonists, has expanded treatment options but simultaneously increased the complexity of decision-making. Providing a PDA before the initial consultation may allow patients more time to process information, reflect on personal preferences, and engage more efficiently with clinicians, ultimately supporting higher-quality decisions.

To address this gap, we undertook a human-centered design process to develop a high-quality digital PDA tailored to AOMs for use in Taiwanese clinical settings. Working collaboratively with health care professionals specializing in weight management, patients, and decision-making stakeholders, our team of industry designers and engineers created and refined OptiWeight, an iPad-based PDA. This study aimed to develop and evaluate OptiWeight by examining its design rationale and assessing its usability, cognitive workload, and subjective user experience.

## Methods

### Study Design

**Figure 1:**
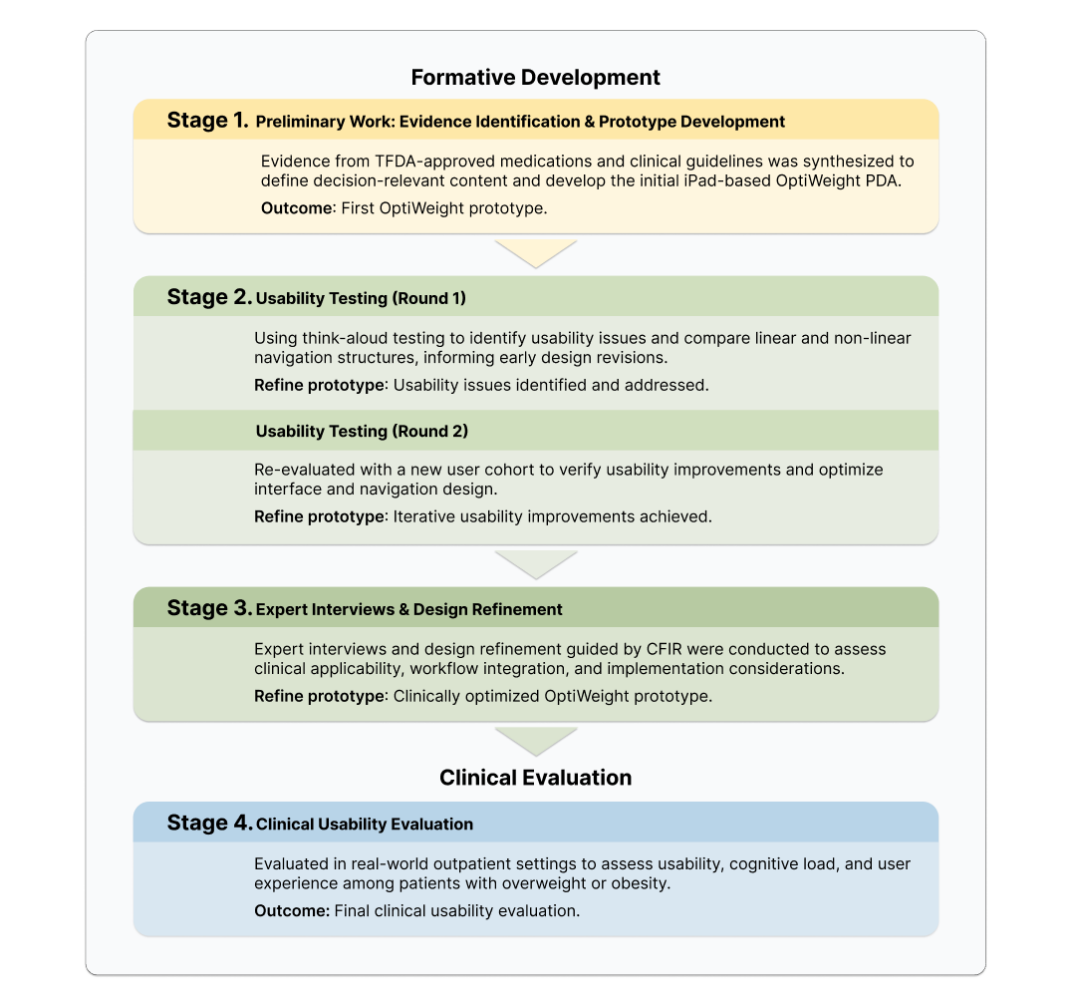
Iterative development and evaluation process of the digital patient decision aid. CFIR: Consolidated Framework for Implementation Research; PDA: patient decision aid; TFDA: Taiwan Food and Drug Administration.

We conducted a user-centered, mixed methods development and evaluation study across 2 medical centers, namely, Taipei Veterans General Hospital and Taipei Medical University Hospital, in Taiwan. The study followed a multicycle iterative design process grounded in the human-centered design framework and the principles outlined in ISO (International Organization for Standardization) 9241-210 [27]. In the context of PDA development, user-centered design entails iterative cycles of understanding end users, developing and refining prototypes, and systematically observing how users interact with these prototypes [28,29]. An overview of the development and evaluation process is presented in Figure 1.

Our development process was informed by established user-centered PDA design methodologies [28,29], guidance from the IPDAS Collaboration [30-32], and the IPDAS reporting checklist [33]. The interdisciplinary research team included a doctoral researcher specializing in SDM (LJW), a clinical expert in obesity management (YJW), and a patient-centered design specialist (MCZ).

Across the overall development process, OptiWeight underwent 3 iterative refinements: (1) after round 1 usability testing (December 2022), usability issues were identified and addressed, with modifications focusing on navigation structure, risk visualization, and onboarding design; (2) after round 2 usability testing (April 2023), further usability improvements were achieved, including finalization of the hybrid navigation architecture and reorganization of medication content; and (3) after expert interviews (stage 3; January-May 2024), the prototype was clinically optimized, with refinements addressing workflow integration, cost display, contraindication filtering, and terminology clarity.

This study adopted a convergent mixed methods design, in which qualitative and quantitative data were collected in parallel at each stage and integrated during interpretation to provide complementary insights into usability and implementation. Qualitative findings were used to explain and contextualize quantitative results, particularly in identifying usability issues and informing iterative design refinements. A consistent inductive content analysis approach was applied across all stages, with stage-specific frameworks used where appropriate to guide interpretation.

### Preliminary Work: Identification of Evidence and Developing the Prototype (Stage 1)

The initial development phase focused on creating OptiWeight, an interactive, iPad-based digital PDA designed to support patients in selecting appropriate AOMs. To inform the content framework, we reviewed the Healthwise decision aid “Obesity: Should I Take Weight-Loss Medicine?” [25] and identified all AOMs approved for weight management by the Taiwan Food and Drug Administration (TFDA) as of 2022. We further conducted targeted literature searches to extract clinical evidence on the efficacy, mechanisms of action, and side effects of these medications [34-40].

In addition, we incorporated risk-prediction information from Taiwan’s Health Promotion Administration [34] and consulted the 2022 Obesity Medicine Association Clinical Practice Statement to define the essential information domains for medication-related decision support. These domains included treatment effectiveness (eg, expected weight loss, metabolic improvement), safety (indications and contraindications), side effects, route of administration, cost considerations, and medication availability [41].

To maintain clarity around the core decision problem, we intentionally excluded content related to lifestyle modification or nutrition counseling, such as exercise guidance or calorie calculations. These strategies are foundational for all patients, regardless of medication use. By excluding them, we aimed to ensure that users recognized the specific decision target—choosing among medication options—rather than conflating medication decisions with broader weight-management choices.

### Usability Testing of the Prototype (Stage 2)

Two rounds of usability testing were conducted to evaluate the OptiWeight prototype and compare 2 information architecture formats—system-controlled navigation and user-controlled navigation.

Participants were recruited through convenience sampling using posters placed on university campuses and community bulletin boards. Interested individuals contacted the research team and were screened for eligibility. Eligible participants were adults aged 20 years or older who were able to operate and view the interface, with assistance if needed. Exclusion criteria included severe visual impairment, cognitive impairment, or inability to provide informed consent.

BMI was not collected during early prototype usability testing (Stage 2) because the focus of this stage was interface usability rather than clinical characterization; consistent with established conventions in human-computer interaction research, general adult samples are appropriate for identifying fundamental usability issues that are likely to persist or be amplified among clinical users [42]. Additionally, omitting BMI reduced participant burden and potential sensitivity related to weight stigma. BMI was subsequently included in the clinical evaluation stage to better describe the target population.

In the first round, initiated in December 2022, 2 separate recruitment posters—1 for each navigation prototype—were posted simultaneously at the same locations with a shared response deadline. Participants self-selected into a group by responding to the respective poster, resulting in unequal group sizes (system-controlled navigation: n=21; user-controlled navigation: n=17). No randomization or stratification by participant characteristics was applied. For round 2, to address the unbalanced allocation in round 1, recruitment was designed to achieve equal group sizes: the first 20 respondents to each poster were enrolled, resulting in balanced groups (n=20 per group). No participant withdrawals were observed during round 1 and round 2 usability testing.

Findings from the first round informed iterative revisions to the prototype, which was subsequently evaluated in a second round of usability testing conducted in April 2023.

Participants were invited to complete tasks using both navigation structures without time restrictions. User interactions were captured through screen recordings and the think-aloud protocol [43]. Following the task session, participants completed a semistructured interview and subsequently filled out the System Usability Scale (SUS) [44] and the NASA Task Load Index (NASA-TLX) [45]. Both the SUS and NASA-TLX were administered online once, immediately after completion of all task activities. SUS and NASA-TLX scores were summarized using descriptive statistics (means and SDs), and cross-stage comparisons are described in the “Statistical Analysis” section. The semistructured interview guide was developed based on established usability evaluation frameworks and iteratively refined during early sessions to improve clarity and relevance.

Qualitative data were deidentified and analyzed using inductive content analysis [46]. Transcripts were first read in full to gain an overall sense of the data, followed by open coding to identify usability-relevant themes. Initial open coding was conducted by 1 researcher (LJW) and independently reviewed by a second researcher (MCZ) to enhance analytical rigor. Coding discrepancies were resolved through discussion until consensus was reached. The research team acknowledged their prior experience in PDA design and usability research and took steps to minimize potential bias through independent coding and consensus discussions. Member checking was not performed, as the data were deidentified before analysis. Usability issues, representative participant quotes, and recommended design modifications were summarized in a structured table.

Both usability rounds included assessments of system- and user-controlled navigation prototypes. Detailed comparative analyses of navigation structures are reported elsewhere [47]. Therefore, in this study, we report aggregated mean values across navigation conditions to illustrate overall usability trends across development stages.

### Expert Interviews and Design Refinement (Stage 3)

Between January and May 2024, we conducted expert interviews at 2 medical centers with health care professionals involved in weight management, including doctors who specialize in obesity medicine, nurses, dietitians and nutritionists, and pharmacists. Eligible participants were adults aged 20 years or older. Eligible health care professionals were identified through purposive sampling: researchers directly invited clinical colleagues with relevant expertise in weight management, and recruitment posters were additionally displayed in clinical areas of the participating medical centers. Experts were invited to evaluate the clarity of medication information, visual and interactive design, and the feasibility of integrating OptiWeight into routine clinical workflows.

To identify user needs and evaluate implementation considerations, we applied the Consolidated Framework for Implementation Research (CFIR) [48,49], which has been widely used to guide implementation of clinical decision support tools (eg, BREASTChoice) [50]. We adapted the 5 CFIR domains—Intervention Characteristics, Outer Setting, Inner Setting, Characteristics of Individuals, and Process—and mapped the resulting themes onto specific design decisions, including interface layout, informational content, interaction patterns, and instructional guidance. This approach allowed us to address design issues unique to an iPad-based PDA for AOMs while systematically examining how individual, organizational, and external contextual factors may influence adoption.

One-on-one interviews were conducted in private rooms within the hospitals and lasted approximately 20-40 minutes. All recruited experts completed the interview, and no participants withdrew or declined to participate. All interviews were audio-recorded, deidentified, and transcribed verbatim for analysis.

After the interview, participants also completed the SUS [44] and the NASA Task Load Index (NASA-TLX) [45] online, providing quantitative measures of perceived usability and cognitive workload. SUS and NASA-TLX scores were summarized using descriptive statistics (means and SDs); cross-stage comparisons are described in the “Statistical Analysis” section.

Interview transcripts were analyzed using inductive content analysis. Initial open coding was conducted by the first author (LJW), who read transcripts in full and identified meaningful segments. A preliminary coding framework (codebook) was developed inductively based on recurring patterns in the data and iteratively refined during analysis. Resulting themes were then organized according to the 5 CFIR domains, which had guided the interview question design. Coding was independently reviewed by a senior researcher with expertise in human-centered design (MCZ) to enhance analytical rigor, and discrepancies were resolved through discussion until consensus was reached. No dedicated qualitative analysis software was used; all data were managed and coded manually.

### Clinical Usability Evaluation (Stage 4)

In February 2025, we conducted a clinical usability evaluation in weight-management outpatient clinics at 2 medical centers. Adults aged 20 years or older with overweight or obesity, that is, a BMI of 24 kg/m^2^ or higher as defined by Taiwan’s guideline [51], who were able to independently operate a digital interface were eligible for participation. Individuals requiring assistance to use the interface or those with impaired consciousness were excluded. Participants were recruited through 2 approaches: attending physicians informed eligible patients during clinic visits, and recruitment posters were displayed in outpatient clinic areas. During their clinic visit, participants interacted with the further refined, finalized version of OptiWeight and subsequently completed the SUS [44] and the NASA Task Load Index (NASA-TLX) [45] in a paper-based format. Both the SUS and NASA-TLX were administered once, immediately after completion of the interaction tasks. SUS and NASA-TLX scores were summarized using descriptive statistics (means and SDs); cross-stage comparisons are described in the “Statistical Analysis” section. A brief semistructured interview (10-15 minutes) was conducted to explore participants’ clinical use experience, perceived cognitive workload, and interaction patterns. All recruited participants completed the study procedures, with no reported withdrawals.

Interview transcripts were analyzed using inductive content analysis. Initial open coding was conducted by one researcher (LJW) and independently reviewed by a second researcher (MCZ) to enhance analytical rigor. Discrepancies were resolved through discussion until consensus was reached. Themes were organized according to Nielsen’s 5 usability heuristics—learnability, efficiency, memorability, error tolerance, and satisfaction—to complement and contextualize the quantitative usability findings. No dedicated qualitative analysis software was used; all data were managed and coded manually.

### Statistical Analysis

Basic demographic data were compared using chi-square tests for categorical variables and independent-samples *t* tests (unpaired and 2-tailed) for continuous variables. Sex (male or female, as assigned at birth) was self-reported and used for demographic characterization throughout the study. To assess the effects of iterative refinements on usability and cognitive workload across the development process, we compared SUS and NASA-TLX scores between stage 2 usability testing (rounds 1 and 2), stage 3 expert interviews, and stage 4 clinical evaluation using 1-way analysis of variance (ANOVA). Before analysis, normality was assessed using the Shapiro-Wilk test and homogeneity of variance using the Levene test. Where assumptions were violated, Welch ANOVA was used as the primary test, and Games-Howell post hoc comparisons were applied. NASA-TLX subscale analyses were treated as exploratory and interpreted descriptively; no correction for multiple comparisons was applied across ANOVAs. All statistical tests were 2-tailed with a significance level of α=.05, and a *P* value <.05 was considered statistically significant. Statistical analyses were conducted using IBM SPSS Statistics (version 29; IBM Corp).

No formal a priori power analysis was conducted. This study followed an exploratory, iterative human-centered design approach in which the primary goal was to identify usability issues and inform design refinements rather than to test predefined statistical hypotheses. Consistent with established conventions in human-computer interaction research, small purposive samples were considered appropriate for usability evaluation [42], and data saturation was not formally assessed, as sample sizes were predefined to support iterative testing and refinement.

All qualitative data—including think-aloud recordings, interview transcripts, and field observations—were complete across all stages; no qualitative data were missing. Regarding quantitative data, no missing data were identified in stage 2 or stage 4. In stage 3, 3 of 18 (17%) participants were excluded from SUS and NASA-TLX analyses due to incomplete questionnaire responses; complete data were therefore available for 15 participants. Little missing completely at random test indicated that the missing data were missing completely at random (*χ*^2^_2_=4.811, *P*=.09), supporting the use of listwise deletion. Given the small number of missing cases and the exploratory nature of the stage, multiple imputation was not performed.

This study was reported in accordance with the GRAMMS (Good Reporting of a Mixed Methods Study; Multimedia Appendix 1) guidelines and the COREQ (Consolidated Criteria for Reporting Qualitative Research; Multimedia Appendix 2) checklist.

### Ethical Considerations

Ethical approval was obtained from multiple institutional review boards across different study phases. Stage 1 involved no human participants and did not require ethical approval. Stage 2 usability testing was approved by the Research Ethics Committee of National Taiwan University (approval number 202206ES059). Stages 3 and 4, conducted at 2 medical centers, were approved by the Institutional Review Board of Taipei Veterans General Hospital (approval number 2024-01-013AC) and the Taipei Medical University-Joint Institutional Review Board (approval number N202403021). All participants provided written informed consent before participation. Upon completing the study procedures, experts received a gift card valued at approximately US $10, and general participants received one valued at approximately US $6.

## Results

### Stage 1: Prototype Development

The initial prototype was developed in Figma (Figma Inc) and presented evidence-based information on available AOMs, associated risks, and potential side effects. Following clinical review by the obesity specialist (YJW), the medication list was refined to include all 3 weight-management medications approved by the TFDA as of 2022—orlistat, naltrexone-bupropion (Contrave), and liraglutide (Saxenda)—as well as semaglutide (Ozempic), which was available during the study period and contains the same active ingredient as semaglutide (Wegovy), a United States FDA–approved medication for weight-loss indications.

Prototype development was led by the interface design specialist (MCZ) and the research team, guided by established visual design principles for health communication [52] and best practices for presenting risk information in decision aids [53,54]. Prior research demonstrates that icon arrays effectively convey statistical risk information—such as natural frequencies (eg, 1 out of 100)—and are particularly beneficial for individuals with lower numeracy skills [55,56]. Moreover, studies by Zikmund-Fisher and colleagues [54] show that anthropomorphic icons (eg, restroom-style human figures) enhance recall of risk information.

Informed by this evidence, the updated prototype incorporated icon-based representations and visualized risk formats to optimize cognitive processing and memory retention. The layout and visual flow were deliberately refined to support comprehension and improve user experience [57,58].

### Stage 2: Usability Testing (Rounds 1 and 2)

#### Overview

Stage 2 involved 2 rounds of usability testing comparing 2 navigation structures: a *system-controlled* prototype, which guided users through content in a fixed sequence, and a *user-controlled* prototype, which allowed flexible navigation across sections.

#### Participant Characteristics of Stage 2 Usability Testing

Across the 2 rounds of stage 2 usability testing, a total of 78 participants were included. In round 1, 38 participants were recruited (21 in the system-controlled navigation group and 17 in the user-controlled navigation group), with a mean age of 26.5 (SD 7.6) years. In round 2, 40 participants were included, with equal allocation to the system- and user-controlled prototypes (n=20 each) and a mean age of 25.1 (SD 6.1) years. Participant characteristics were comparable across rounds in terms of age, sex, and education level (Table 1).

**Table 1:** Participant characteristics of prototype usability testing (rounds 1 and 2).

Characteristics			Round 1 (n=38)										Round 2 (n=40)											
			Overall		System-controlled navigation group (n=21)		User-controlled navigation group (n=17)		*P* value		Overall				System-controlled navigation group (n=20)			User-controlled navigation group (n=20)			*P* value			
**Age (years** **), mean (SD)**			26.5 (7.6)		27.6 (9.8)		25.0 (3.1)		.30		25.1 (6.1)				23.25 (1.7)			26.85 (8.1)			.06			
**Sex,** **n (%)**									>.99												.11			
	Male	25 (66)		14 (67)		11 (65)				18 (45)				12 (60)			6 (30)							
	Female	13 (34)		7 (33)		6 (35)				22 (55)				8 (40)			14 (70)							
**Education level,** **n (%)**									.48												>.99			
	Bachelor	29 (76)		15 (71)		14 (82)				27 (68)				14 (70)			13 (65)							
	Graduate or above	9 (24)		6 (29)		3 (18)				13 (32)				6 (30)			7 (35)							

#### Usability and Cognitive Workload of Stage 2 Usability Testing

In *usability testing round 1*, usability scores remained in the mid-range for both navigation structures. The user-controlled prototype yielded an SUS score of 59.4 (SD 18.3), while the system-controlled prototype scored 61.4 (SD 20.0). NASA-TLX scores indicated substantial workload across conditions, with the user-controlled structure scoring 45.1 (SD 12.7) and the system-controlled structure scoring 36.5 (SD 20.0). Variability in responses was pronounced, suggesting divergent user experiences: while some participants found the interface intuitive, others reported considerable difficulty. Despite these challenges, most participants expressed positive attitudes toward OptiWeight and felt it improved their understanding of medication options. However, many continued to experience confusion during navigation.

In *usability testing round 2*, the user-controlled prototype received an SUS score of 66.4 (SD 16.4), while the system-controlled prototype scored 66.1. NASA-TLX scores indicated substantial perceived workload, with the user-controlled prototype scoring 46.8 (SD 11.4) and the system-controlled prototype scoring 35.5 (SD 12.1). Similar to round 1, usability ratings showed wide variability, with some participants reporting strong ease of use and others expressing significant difficulty. Participants also described notable mental, physical, temporal, and emotional demands during task completion. Despite these challenges, participants generally responded positively to OptiWeight and felt it improved their understanding of medication options, although aspects of the navigation remained confusing for some.

#### Summary of Stage 2 Usability Testing

In usability testing round 1, participants preferred visualized risk information, particularly bar charts (28/38, 74%). However, brief explanatory text often left users uncertain about the meaning of the graphics. Many recommended pairing visualizations with numerical values to improve interpretability. Participants also requested medication images before detailed descriptions, as visual cues supported understanding of administration methods and improved memory of treatment options. Several participants perceived the medication descriptions as overly brief and recommended adding indications, dosage instructions, and precautions.

A participant with color vision deficiency noted difficulty distinguishing between light-blue and gray icons in the risk displays. Preferences regarding information architecture were mixed: 19 out of 35 (54%) favored the user-controlled structure due to ease of comparison and flexible navigation, although increased information density and nonsequential flow were noted as drawbacks. Conversely, the system-controlled structure reduced cognitive load and provided helpful progress indicators and navigation cues, but made side-by-side comparison difficult and required frequent back-and-forth navigation.

Observational data identified several usability issues: risk and side effect dropdown menus were frequently overlooked; chronic disease items lacked hierarchical separation from the “Next” button; icon arrays were not visually distinguished from text; some participants misunderstood the function of the “Decision” button; and users were uncertain whether disease-related fields were required if they had no chronic conditions. Certain technical medical terms were considered difficult to understand (eg, “type 2 diabetes”), prompting requests for brief explanations. Participants also suggested a short introductory tutorial to reduce initial cognitive load.

In usability testing round 2, interview findings indicated that 27 out of 40 (68%) participants preferred the user-controlled prototype, citing greater flexibility, faster access to desired content, and the ability to view multiple pieces of information on a single page. However, participants also noted drawbacks, including high information load and the absence of progress cues, which made it difficult to determine the end point of the task. The system-controlled structure was valued for its stepwise, guided flow that facilitated deeper comparison of treatment details. Nonetheless, participants reported that the mandatory sequencing resulted in repetitive reading and cumbersome navigation, reducing satisfaction.

Participants continued to express a strong preference for visualized risk information, particularly numerical risk values presented in graphical formats. However, visualizations alone were insufficient; users still relied on accompanying text and numerical values to fully interpret medical information. Medication-related visuals—such as images depicting appearance and dosage form—were viewed as important components of a PDA. Participants explained that medication names were difficult to remember, whereas visual cues supported recall and understanding of administration routes. Given the diversity of users, participants emphasized the need for color choices that accommodate individuals with color blindness or color vision deficiency.

Findings also suggested that information architecture influences users’ risk perception and comprehension. Participants prioritized rapid access to the information they cared about most, and restrictive navigation sometimes hindered understanding, possibly due to reduced attention or frustration. Based on these insights, the final version of OptiWeight adopted a hybrid architecture: a guided system-controlled structure for overall flow, combined with an open, user-controlled layout for medication-specific content to support flexible comparison (Table 2).

**Table 2:** Usability test (round 2) findings and resulting design modifications.

Issue^a^ and illustrative quotes			Modification (with screenshot reference)
**Navigation structure**			
	“It’s faster, more flexible, and I can see more info on one page.” [User-controlled navigation] “I don’t know where the end is—there’s no progress guide.” [User-controlled navigation] “It’s guided and detailed, but too repetitive and frustrating to go back and forth.” [System-controlled navigation] “I like this page where I can see the introduction of all four medications at once, but afterward I can only view one medication at a time and cannot compare their pros and cons simultaneously.” [System-controlled navigation] “Before using this tool, I had no knowledge of these medications. By the time I reached later pages, I had already forgotten the names and details of the earlier ones.” [System-controlled navigation] “I want to quickly find information about medication side effects, but the current sequence forces me to go through risk information first, which does not help me answer my question.” [System-controlled navigation] “There are too many ‘Next’ buttons, and it becomes frustrating.” [System-controlled navigation]	We adopted a hybrid navigation model that retains the progress indicators from the system-controlled structure while allowing free user-controlled browsing within the medication pages. We also rearranged the browsing sequence by moving the medication overview comparison before the individual medication descriptions, enabling users to gain an initial understanding before reviewing detailed therapeutic information.	
**Drug recognition and information**			
	“I want to quickly know which medication results in the greatest weight reduction, but I can’t find it.” “This says the medication leads to 9.3% annual weight loss, but if I only use it for one month, will I get the same effect?”	We added a line chart showing expected weight-loss effects over time for each medication and incorporated weight-loss outcomes directly into the medication summary pages.	
**User guidance/onboarding**			
	“The instruction section at the beginning is too long.” “If this is my second time using the tool, I don’t need to read the tutorial again.”	We shortened the onboarding tutorial and added a Skip button for returning users.	
**Readability and layout**			
	“The medication explanation page contains too much text—it’s long and difficult to read.”	We reorganized the text in the pop-up window for detailed medication information by dividing indications, dosage, side effects, contraindications, and precautions into 3 structured sections. We also reformatted the “risks and side effects” pop-up into 2 grouped content blocks to improve readability.	
**Features and interactivity**			
	“The medication suggests it is only recommended for BMI ≥ 30, but I don’t know what my BMI is.” “The question-mark icon on the medication details pop-up made me think it was a system error message.”	We added a BMI results page that calculates the user’s BMI based on the basic information they entered. We changed the question-mark icon on the medication details pop-up to an ‘i’ (information) symbol to better convey its purpose.	

^a^Issues are derived from think-aloud observations and interviews, and those from round 1 that did not reappear were considered resolved. Round 2 captured both the validation of prior modifications and the emergence of new usability concerns.

### Stage 3: Expert Interviews and Design Refinement

#### Participant Characteristics

A total of 18 health care professionals specializing in weight management, with an average of 16.1 years of clinical practice experience (SD 11.0 years), participated in and completed the expert interviews. The sample included 12 (67%) physicians/surgeons, 1 (6%) nurse, 1 (6%) pharmacist, and 4 (22%) dietitians and nutritionists. Participants were mainly female (n=11, 61%); all had a bachelor’s degree or higher and a mean age of 40.8 (SD 11.2) years (range 27-57 years; Table 3).

**Table 3:** Characteristics of health care professionals specializing in weight management (in stage 3 expert interviews) and patients (in stage 4 clinical evaluation).

Characteristics			Participants				
			Health care professionals (n=18)		Patients (n=78)		
Age (years), mean (SD)			40.8 (11.2)		45.0 (11.1)		
**Sex, n (%)**							
	Male	7 (39)		33 (42)			
	Female	11 (61)		45 (58)			
**Education level, n (%)**							
	Junior high	0 (0)		1 (1)			
	High school	0 (0)		9 (12)			
	College	0 (0)		9 (12)			
	Bachelor	11 (61)		32 (41)			
	Graduate or above	7 (39)		27 (35)			
**Profession, n (%)**							
	Physician/surgeon	12 (67)		N/A^a^			
	Nurse	1 (6)		N/A			
	Pharmacist	1 (6)		N/A			
	Dietitian and nutritionist	4 (22)		N/A			
**BMI (kg/m^2^), n (%)**							
	Overweight (24-26.9)	N/A		9 (12)			
	Mild obesity (27-29.9)	N/A		15 (19)			
	Moderate obesity (30-34.9)	N/A		38 (49)			
	Severe obesity (≥35)	N/A		16 (21)			

^a^N/A: not applicable.

#### Usability and Cognitive Workload of Stage 3 Expert Interviews

Experts reported a mean SUS score of 77.8 (SD 9.4), corresponding to the “good” usability range, indicating that OptiWeight was generally perceived as intuitive and user-friendly. The overall cognitive workload was moderate, with a mean NASA-TLX score of 28.1 (SD 11.3). Of the 18 obesity specialists who participated in the design refinement sessions, complete SUS and NASA-TLX data were available for 15 participants.

#### Summary of Stage 3 Expert Interviews

Using the 5 CFIR domains, we synthesized the perspectives of 18 obesity specialists. Overall impressions were positive, with experts providing an average rating of 8.2 (on a 1=poor to 10=excellent scale). They described OptiWeight as visually appealing, intuitive to use, and effective in presenting visual and textual information that supports patient understanding of medication options. However, several refinements were identified under the *Intervention Characteristics* domain. Experts recommended restructuring lengthy instructional pages into embedded, stepwise guidance with clear status cues; revising the “no treatment” option to “no medication treatment/lifestyle modification”; explicitly noting that liraglutide (Saxenda) should be discontinued if less than 5% weight loss is achieved after 6 months; highlighting the social implications of Orlistat’s oily stools; and clearly labeling semaglutide (Ozempic) as off-label or removing it from the selections. They also recommended organizing medications by administration route (oral versus injection) and expanding cost information to include monthly and full-course expenses. To reduce cognitive burden, experts encouraged replacing technical terminology with more accessible language.

Regarding visual design, experts noted that icon arrays representing treatment effects might be misinterpreted as risks and recommended adding percentage labels or using bar or pie charts. For line graphs, they suggested simplifying the display by reducing the number of lines, removing the placebo curve, and directly annotating key time points to improve interpretability.

Under the *Outer Setting* and *Inner Setting* domains, experts recognized that digital SDM tools align with national policy directions and could enhance institutional visibility. They also emphasized the importance of incorporating local contextual considerations, including Taiwan’s BMI criteria (overweight: 24≤BMI<27 kg/m^2^; obesity: BMI≥27 kg/m^2^) [51], out-of-pocket medication costs under the National Health Insurance system, completeness of pharmaceutical formularies, Ministry of Health and Welfare education requirements, and copyright considerations. Organizational challenges included the costs and depreciation of tablet devices, privacy and Wi-Fi reliability, and variation in drug availability across hospitals. Experts stressed that the tool should reduce—not increase—clinician workload and should be operable by nurses or case managers without conflicting with existing electronic medical record workflows.

Within the *Characteristics of Individuals* domain, experts highlighted varied needs across professional roles (eg, dietitians often engage after medication selection) and noted that older adults or patients with low digital literacy may struggle with line graphs or icon arrays. From their clinical experience, patients most frequently prioritize cost, expected effectiveness, and administration route. Under the *Process* domain, experts recommended enabling patients to access OptiWeight before or during clinic visits using a QR code, with shared tablet viewing during consultations. They suggested providing printable or PDF summaries for home review, along with basic training for nurses and case managers. Evaluation metrics should capture both patient and clinician satisfaction as well as time burden. Experts also emphasized the need for a mechanism to update medication information regularly and suggested expanding the tool to include lifestyle, physical activity, and surgical options in the future, with potential customization by age or comorbidities. Representative quotes and design modifications are summarized in Table 4.

**Table 4:** Expert interviews, insights, and resulting design modifications. This table lists issues that led to concrete design changes.

Issue and illustrative quotes		Modification (with screenshot reference)
**Visualization of risk information**		
	“Repetitive information; excessive content on the page” “It looks like showing risk instead of treatment effect.”	Removed duplicated charts describing risks and side effects, retaining only the icon array. Reframed the section title from “Risk” to “Medication Effects,” adding a percentage label such as “Risk reduction: X%” to clarify that the visualization represents the benefits of treatment.
**Drug recognition and information**		
	“Line graphs contained too many lines, impairing comprehension; unclear purpose of the placebo curve” “Y-axis scale too long; users wanted clearly marked treatment duration and effect size” “It only says the unit price. Patients don’t know how long one box lasts or how much per month.” “Patient would like to know the total cost of one full treatment course, not just per injection.” “Experts recommended adding dosing frequency, dosage, and storage instructions” “Need to clarify Orlistat’s oily stool and its social impact” “Saxenda should specify that treatment should stop if <5% weight loss after 6 months”	Removed the placebo curve from line graphs to improve readability. Shortened the y-axis time scale: replaced 52-week display with 4 nodes (3, 6, 9, and 12 months). Added clickable time point buttons, allowing users to view expected weight-loss percentage and total projected cost. Added monthly cost estimates in addition to unit prices. Expanded the medication details pop-up to include dosing frequency, dosage (for adults and adolescents), storage requirements, and an explicit stop-treatment rule. Clarified that Orlistat’s oily stool may affect social activities.
**User guidance/onboarding**		
	“I wasn’t sure if I had entered the real operation or was still in the tutorial.” “The guidance pages felt too long—I got lost halfway.”	Converted the tutorial to embedded (inline) guidance, shortening the number of pages and removing nonessential instructional screens.
**Features and interactivity**		
	“It feels like the system just abandons me when I choose no treatment.” “This PDA focuses on medications; the title should reflect that more clearly.” “Some patients care a lot about specific side effects. It would be helpful if the system allowed comparison of medications with the same side effect.” “Recommend adding contraindication filtering (BMI, age, comorbidities, indications).” “It feels like the system just abandons me when I choose no treatment.”	Updated the tool title from “Weight-Loss Treatment” to “Weight-Loss Medication Decision Aid” for clarity. Added a side effect comparison feature for medications sharing the same adverse events. Implemented intelligent contraindication filtering, marking medications unsuitable for the patient with grayscale labels. Reworded “No treatment” to “Not using medication for now (lifestyle modification)” to improve clarity and reduce negative tone.

### Stage 4: Clinical Evaluation

#### Content and Workflow of the Final OptiWeight

**Figure 2:**
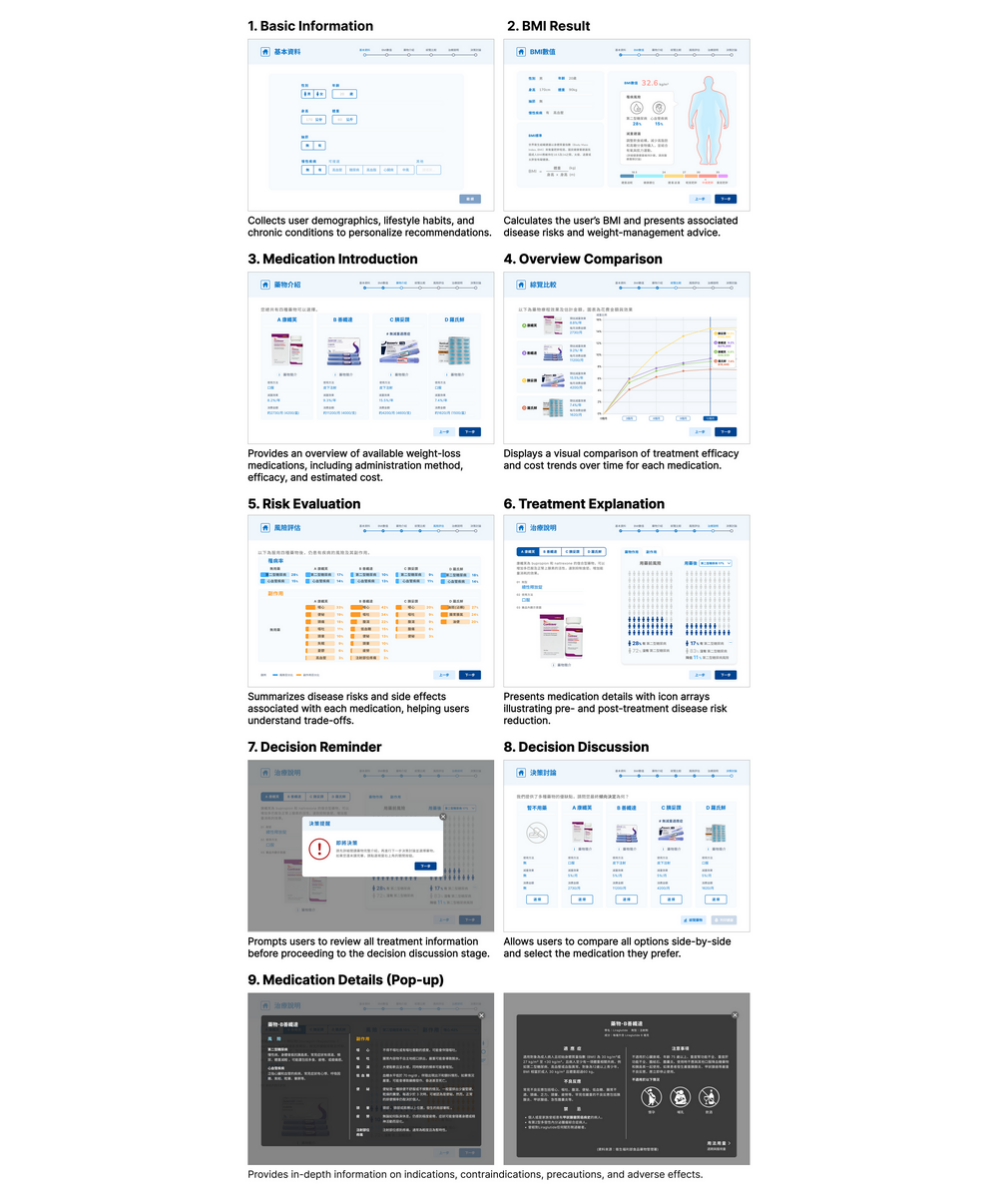
Layouts of the finalized OptiWeight patient decision aid evaluated in clinical usability testing.

Following the previous stages, OptiWeight was refined and finalized as an interactive iPad-based PDA titled “Antiobesity Medication Decision Aid,” displayed in Traditional Chinese to ensure regional applicability. The tool guides users through 6 sequential components that provide comparative information on AOMs, including treatment efficacy, side effects, administration routes, and out-of-pocket costs (Figure 2).

#### Participant Characteristics of Stage 4 Clinical Evaluation

A total of 78 patients with overweight or obesity receiving weight-management care participated in the clinical usability evaluation. Participants had a mean age of 45.0 (SD 11.1) years (range 22-74 years), were mainly female (n=45, 58%), had a college-level education or higher, and 54 out of 78 (69%) were classified as having moderate to severe obesity (BMI ≥30 kg/m^2^; Table 3).

#### Usability and Cognitive Workload of Stage 4 Clinical Evaluation

The overall mean SUS score was 73.65 (SD 14.20), indicating “good” usability for the final OptiWeight prototype. The mean NASA-TLX score was 16.69 (SD 14.44), suggesting low perceived workload in the clinical context. Among the subscales, *mental demand* and *effort* were rated comparatively higher (mean 22-29), whereas *physical demand*, *temporal demand*, and *frustration* remained low to moderately low. Collectively, these findings support that the final version of OptiWeight demonstrates acceptable usability and operational feasibility in outpatient practice.

#### Summary of Stage 4 Clinical Evaluation

##### Qualitative Evaluation Using Nielsen’s Usability Heuristics

To enrich the quantitative findings, qualitative interviews were analyzed using Nielsen’s 5 usability heuristics—learnability, efficiency, memorability, error tolerance, and satisfaction. This analysis examined whether the final OptiWeight prototype met real-world clinical usability needs.

##### Learnability

Participants demonstrated varying familiarity with AOM therapy. First-time users often needed clarification regarding medical terminology and the purpose of the tool. Participants emphasized the value of tooltips, embedded definitions, and clearer contextual guidance at the outset (eg, whether the tool is intended for self-learning or to support clinician-patient dialogue). These findings suggest that OptiWeight supports learnability with minimal guidance but could further benefit from concise instructional prompts and plain-language explanations.

##### Efficiency

Although participants appreciated the logical structure and clean layout, many sought faster access to key decision-relevant information. Suggestions included consolidating medication attributes and risk information into a single screen, enabling automated conversion of weight-loss percentages into kilograms, and ranking medication options by cost-effectiveness. Visualizations combining cost and efficacy were perceived as cognitively demanding, and separating these data elements was recommended to improve clarity.

##### Memorability

Participants described the interface as memorable due to its visual organization and intuitive iconography. However, small font sizes posed challenges for older adults. The ability to revisit information—via a dedicated “Back” button or a summary page—and to print a summary for later review with family or clinicians was viewed as essential for reinforcing comprehension and memory.

##### Error Tolerance

Technical errors were minimal, yet some participants recommended that all medication options remain visible with explicit explanations (eg, “Not recommended because BMI <27 kg/m^2^”). The term “decision discussion” on the final screen was perceived as overly formal; alternatives such as “treatment preference summary” were preferred.

##### Satisfaction

Overall satisfaction was high. Participants described OptiWeight as clear, modern, and highly practical for understanding treatment options within minutes. Visual risk displays—especially those illustrating improvements in metabolic or cardiovascular risk—were seen as motivating. Participants recommended adding more engaging visuals (eg, brief animations or before-and-after illustrations) and suggested integrating lifestyle guidance or goal-setting features to contextualize medication effects within personal health goals.

Overall, the clinical evaluation demonstrated that OptiWeight was well accepted by patients and supported effective understanding of treatment options in real-world outpatient settings. Participants viewed the tool as intuitive and informative and provided actionable recommendations for enhancing accessibility and clarity across diverse patient populations. These findings underscore the clinical usability and practical feasibility of the final OptiWeight prototype.

### Usability and Cognitive Workload Across Evaluation Stages

Across the iterative development process, the app interface was progressively refined between stage 2 usability testing (rounds 1 and 2), stage 3 expert interviews, and stage 4 clinical evaluation, with corresponding increases in usability and decreases in perceived workload (Table 5).

**Table 5:** System Usability Scale and NASA-Task Load Index scores across usability testing, expert evaluation, and clinical evaluation stages^a^.

Measure			Usability testing round 1 (n=38)		Usability testing round 2 (n=40)		Expert interviews (n=15)		Clinical evaluation (n=78)
System Usability Scale total score, mean (SD)			60.53 (19.04)		66.25 (14.68)		77.83 (9.44)		73.65 (14.20)
**NASA-Task Load Index total score, mean (SD)**			40.35 (17.49)		41.13 (12.93)		28.11 (11.31)		16.69 (14.44)
	Mental demand	64.21 (22.50)		63.25 (19.13)		39.67 (24.67)		22.50 (23.60)	
	Physical demand	19.47 (21.68)		24.50 (17.68)		14.33 (14.25)		9.68 (11.99)	
	Temporal demand	28.42 (25.74)		32.50 (23.83)		25.33 (24.67)		10.77 (13.51)	
	Performance	48.68 (28.21)		50.00 (21.12)		27.33 (25.97)		28.59 (26.16)	
	Effort	52.11 (23.73)		49.50 (18.25)		48.33 (26.37)		28.59 (26.35)	
	Frustration	29.21 (26.75)		27.00 (25.34)		13.67 (16.53)		13.27 (18.55)	

^a^Round 1 and Round 2 scores are averaged across navigation prototypes (system- and user-controlled navigation).

Assumption checks indicated violations of normality (Shapiro-Wilk tests) and heterogeneity of variances for several variables; therefore, Welch ANOVA with Games-Howell post hoc comparisons was applied.

SUS scores demonstrated a clear upward trend, increasing from usability testing round 1 to round 2 and peaking during the expert evaluation stage (mean 77.83), while remaining relatively high during clinical evaluation (mean 73.65; Table 5). Welch ANOVA revealed a significant effect of evaluation stage (Welch *F*_3,58.13_=8.54, *P*<.001, η^2^=0.13; Table 6). Games-Howell post hoc comparisons indicated that round 1 scores were significantly lower than expert interview scores (mean difference −17.31, *P*<.001) and clinical evaluation scores (mean difference −13.13, *P*=.002). Round 2 scores were also significantly lower than expert interview scores (mean difference −11.58, *P*=.007) and did not differ significantly from clinical evaluation scores (mean difference −7.40, *P*=.05). No significant differences were observed between round 1 and round 2 (*P*=.45) or between expert interviews and clinical evaluation (*P*=.49).

**Table 6:** One-way analysis of variance results for System Usability Scale and NASA-Task Load Index scores across evaluation stages (round 1, round 2, expert interviews, and clinical evaluation)^a^.

Measure			*F* test (*df*)		*P* value		η^2^
System Usability Scale total score			8.46 (3, 167)		<.001		.13
**NASA-Task Load Index total score**			35.25 (3, 167)		<.001		.39
	Mental demand (NASA_1)	43.81 (3, 167)		<.001		.44	
	Physical demand (NASA_2)	8.31 (3, 167)		<.001		.13	
	Temporal demand (NASA_3)	12.73 (3, 167)		<.001		.19	
	Performance (NASA_4)	9.56 (3, 167)		<.001		.15	
	Effort (NASA_5)	11.67 (3, 167)		<.001		.17	
	Frustration (NASA_6)	6.34 (3, 167)		<.001		.10	

^a^Groups included round 1 (n=38), round 2 (n=40), expert interviews (n=15), and clinical evaluation (n=78). Reported *F* test values and η^2^ correspond to standard 1-way ANOVA. Welch ANOVA was additionally conducted to account for violations of homogeneity of variance, and consistent results were observed. Post hoc comparisons were conducted using Games-Howell tests.

NASA-TLX total scores exhibited a progressive decline across iterations (Table 5). Welch ANOVA revealed a significant effect of evaluation stage (Welch *F*_3,55.36_=35.08, *P*<.001, η^2^=0.39; Table 6). Games-Howell post hoc tests showed that both round 1 and round 2 had significantly higher workload than the expert evaluation stage (round 1: mean difference 12.24, *P*=.02; round 2: mean difference 13.01, *P*=.005) and the clinical evaluation stage (round 1: mean difference 23.66, *P*<.001; round 2: mean difference 24.44, *P*<.001). Workload remained significantly higher in the expert evaluation stage than in the clinical evaluation stage (mean difference 11.42, *P*=.01). No significant difference was observed between round 1 and round 2 (*P*=.996). Similar trends were observed across NASA-TLX subscales, particularly for mental demand, effort, and temporal demand, suggesting a substantial reduction in cognitive workload across evaluation stages.

## Discussion

### Principal Findings

Using a human-centered design approach and iterative refinement, this study developed and validated OptiWeight, a PDA designed to support AOM decisions in Taiwanese clinical settings. Across the development cycle, usability increased across stages: SUS scores rose from mid-range levels during early prototype testing to good usability in the expert evaluation stage and remained high during real-world clinical use. Concurrently, cognitive workload decreased markedly, with NASA-TLX scores declining from the prototype stage to expert testing and clinical evaluation, suggesting progressive improvements associated with iterative refinement. These findings are best interpreted as reflecting prototype maturation across development stages; however, variations in participant populations—including general adults, clinical experts, and patients—may also have influenced the observed outcomes.

Qualitative findings further elucidated why these usability improvements occurred. Participants reported that combining numerical and visual formats enhanced comprehension, while a hybrid navigation structure—balancing guided flow with flexible browsing—supported more efficient information seeking and reduced cognitive load. The final clinical assessment suggested that OptiWeight is intuitive and acceptable to users, and may support informed patient-clinician discussions about AOMs from a usability perspective.

### Interpretation and Comparison With Prior Work

Most existing PDAs for weight management have focused on decisions regarding bariatric surgery or comprehensive weight-management programs. For example, Arterburn et al [23] developed a tool to help patients decide whether to pursue bariatric surgery, whereas Moore et al [21] compared intensive lifestyle management with surgical pathways. Lee and Wu [24] designed a PDA to support choices among different bariatric procedures. Chu et al [59] extended PDA applications further upstream by using an artificial intelligence–based system to guide patients among 3 broad weight-management routes: self-directed lifestyle adjustments, dietitian-assisted programs, or physician-led treatment. By contrast, OptiWeight addresses a distinct and underexplored decision point—choosing among multiple medication options. As new-generation medications such as glucagon-like peptide-1 receptor agonists increasingly diversify available choices, patients must weigh differences in efficacy, safety profiles, routes of administration, and out-of-pocket costs. OptiWeight fills this gap by providing a structured, localized comparison framework tailored to Taiwanese clinical practice.

Patient preferences are known to play a central role in adherence and clinical outcomes in weight management [8]. Decisions between obesity medications are influenced by perceived effectiveness, access to information, safety concerns, and the practicality of use [10]. To our knowledge, OptiWeight is the first PDA designed specifically to support preference-sensitive decisions among medication options, enabling patients to systematically evaluate trade-offs and clarify personal priorities—such as maximizing weight-loss efficacy versus minimizing gastrointestinal side effects—potentially supporting more informed, value-concordant treatment choices.

While prior PDA research has primarily emphasized single-point clinical evaluations of tool effectiveness [23], only a few studies have detailed how user feedback is translated into design refinements across iterative cycles. Although Moore et al [21] and Lee and Wu [24] reported aspects of their development processes, published descriptions focused mainly on feasibility and acceptability rather than the iterative mechanisms by which interface design and content were adjusted. By contrast, our study used a multistage, iterative human-centered design process that systematically linked prototype testing, expert interviews, and clinical usability assessment. This approach provides a transparent and reproducible development pathway for digital PDAs and highlights the importance of usability and workflow integration as parallel design goals, addressing a gap in the literature that often privileges outcome evaluation over process evidence. This aligns with user-centered design principles emphasizing continuous cycles of understanding, designing, evaluating, and refining to ensure that content and interaction patterns match real-world contexts and users’ mental models [28,29].

Using the widely accepted SUS [60,61], OptiWeight demonstrated substantial usability improvement across iterations—from an early score of 60.53 to 73.65 in the final clinical evaluation. According to established SUS benchmarks, scores above 70 represent a “passable product” [44], scores above 71.4 indicate “good” usability [62], and scores above 80.8 reflect “excellent” usability [63]. The improvement observed across rounds is consistent with the iterative, user-centered refinement process and highlights SUS as a practical index for guiding PDA development toward clinical adoption and user satisfaction [64].

We also evaluated cognitive workload using the NASA Task Load Index (NASA-TLX) [45]. Prior work indicates that typical mean NASA-TLX scores for cognitive and medical tasks fall below 54 [65]. OptiWeight’s final clinical score of 16.69 reflects a substantially lower workload, which may be associated with interface refinements, although differences in participant characteristics across stages may also have contributed to this trend. Cognitive Load Theory [66] distinguishes intrinsic, extraneous, and germane cognitive load; our findings highlight the importance of minimizing extraneous load attributable to poor interface design. By streamlining information presentation, simplifying navigation structure, and reducing unnecessary cognitive effort, OptiWeight is designed to help users focus on essential content without overwhelming their working memory capacity.

User-testing findings further informed several concrete design choices. Initially, we explored system-controlled versus user-controlled navigation structures but ultimately converged on a hybrid navigation architecture—a system-controlled backbone providing predictability and orientation cues, combined with flexible, user-controlled browsing within medication sections. This approach aligns with established information architecture principles, including focused navigation, multiple classification, and meaningful choice [64,67,68], while supporting chunking to accommodate limited working memory capacity [69,70].

In visual risk and benefit communication, OptiWeight integrates numeric and icon-based representations, consistent with evidence suggesting that enclosing visual elements within structured frames enhances comprehension [71] and that highlighting treatment-placebo differences reduces users’ mental computation demands and cognitive load [57,72]. Our iterative refinements included improving label consistency, ensuring equivalent visibility across medication categories, adopting plain-language descriptions [54], and implementing color-contrast accessibility considerations for users with color vision deficiencies [71]. Universal back-navigation and error-recovery pathways were also strengthened to reduce interaction uncertainty and enhance learnability [73].

Finally, the effectiveness of PDAs heavily depends on cultural relevance and contextual fit [74]. Without adequate cultural adaptation [75], tool applicability across populations is limited. IPDAS similarly emphasizes localization of language and content [30]. Existing weight-management PDAs reflect their respective health system contexts—for example, Arterburn et al’s [23] tool incorporates elements specific to the US insurance system, while Moore et al’s [21] PDA targets adolescents, whose developmental and family decision-making dynamics differ markedly from adults. In Taiwan’s unique context—characterized by a single-payer national health insurance system, universal out-of-pocket costs for AOMs, and distinct clinical workflows—local adaptation is essential. Expert interviews highlighted strong clinical demand and perceived market potential for such tools; transparent cost presentation, clear availability labeling, and workflow integration across multidisciplinary roles were viewed as key factors for adoption and equity.

### Strengths and Limitations

This study has several strengths, including the use of a multistage human-centered design process, triangulation of quantitative and qualitative data, and validation across prototype, expert, and clinical settings. However, several limitations should be noted. First, to maintain temporal consistency during development, we did not include semaglutide (Wegovy) or tirzepatide (Mounjaro), which were approved by the TFDA during or after the study design and implementation period. Second, this work focused on usability and clinical feasibility; decision quality outcomes—such as patient knowledge, decisional conflict, and value concordance—were not assessed, nor were medium- or long-term clinical end points. Third, different participant populations were involved at each stage, and observed differences in usability and cognitive workload may partially reflect population characteristics rather than solely the effects of iterative design refinements. Future studies should consider randomized or staged implementation designs that incorporate broader evaluation metrics, including decision quality, consultation time, and team workload, alongside longitudinal behavioral and clinical outcomes such as medication adherence, weight trajectories, metabolic indicators, medication switching or discontinuation events, and sustained lifestyle changes. In addition, expanding support for multiple device types and integrating OptiWeight with electronic health record systems would further clarify its scalability, interoperability, and potential to promote equitable access in real-world settings.

### Conclusions

This study developed OptiWeight, a digital PDA with a patient-centered, interactive design to support adults with overweight or obesity in making preference-sensitive choices among AOMs. By presenting locally relevant, easy-to-understand comparisons of medication efficacy, side effects, administration routes, and out-of-pocket costs within the constraints of routine outpatient care, OptiWeight provides structured and accessible support for comparing AOM options, which may facilitate patient understanding and consideration of treatment choices. Through multicycle design and validation—including iterative prototype testing, expert interviews, and on-site clinical evaluation—we operationalized international PDA development principles into a functional interactive tool and workflow, offering a replicable blueprint for future digital PDA development. Qualitative findings further indicated that visualized risk information was most effective when paired with numerical and contextual explanations, and that hybrid navigation helped balance cognitive load with flexible comparison. Future work will focus on evaluating real-world implementation, creating alternative formats to enhance accessibility, and establishing a sustainable update strategy to support equitable, evidence-based AOM services in Taiwan.

## Data Availability

The datasets generated and analyzed during this study are not publicly available due to ethical and privacy considerations, as they may contain potentially identifiable information. Deidentified data, including quantitative datasets and qualitative coding materials, are available from the corresponding author (MCZ) upon reasonable request.

## Appendix

Multimedia Appendix 1 GRAMMS (Good Reporting of a Mixed Methods Study) checklist.

Multimedia Appendix 2 COREQ (Consolidated Criteria for Reporting Qualitative Research) checklist.

## Abbreviations

ANOVA: analysis of variance
AOM: antiobesity medication
CFIR: Consolidated Framework for Implementation Research
COREQ: Consolidated Criteria for Reporting Qualitative Research
GRAMMS: Good Reporting of a Mixed Methods Study
IPDAS: International Patient Decision Aid Standards
ISO: International Organization for Standardization
PDA: patient decision aid
SDM: shared decision-making
SUS: System Usability Scale
TFDA: Taiwan Food and Drug Administration
