# Supplementary material for: Digital Patient Decision Aid for Antiobesity Medications: Mixed Methods Study of Human-Centered Design and Usability Evaluation
Source: J Med Internet Res. 2026 May 15;28:e89428. doi: 10.2196/89428 (PMC13221622; doi:10.2196/89428)
Supplement: Multimedia Appendix 1 [file jmir_v28i1e89428_app1.docx]

# Good Reporting of a Mixed Methods Study (GRAMMS) Checklist

*Reference: O'Cathain A, Murphy E, Nicholl J. The quality of mixed methods studies in health services research. J Health Serv Res Policy. 2008;13(2):92–98.*

Design: Convergent parallel mixed methods; 4-stage human-centered design framework; study period August 2022–November 2025.

| **Guideline Section** | **Section(s)** | **Description / Reporting Details** |
| --- | --- | --- |
| 1. Justification for using a mixed methods design to address the research question | Section 1, 2.1 | The manuscript justifies the convergent mixed-methods design in the Introduction and Methods (Section 2.1). Quantitative measures (SUS, NASA-TLX) alone cannot capture the nuanced usability problems or HCPs' implementation perceptions, while qualitative data alone cannot provide generalisable benchmarks. The authors explicitly state that integration of both strands enables a more complete understanding of usability, user experience, and clinical adoption of the PDA. |
| 2. Description of the mixed methods design: what was the purpose, what was the priority of each method, and what was the sequence of the methods | Section 2.1–2.5 | Design: Convergent parallel mixed-methods embedded within a 4-stage human-centered design (HCD) framework (August 2022–November 2025). Purpose: to develop and evaluate the OptiWeight PDA iteratively. Priority: qualitative and quantitative strands given equal priority (QUAL + QUAN). Sequence: Stages 1–4 are sequential in the development cycle, but within each evaluation stage (Stages 2–4) qualitative and quantitative data were collected concurrently and integrated at interpretation. Stage 1 = needs assessment (qualitative); Stage 2 = alpha usability testing (QUAL + QUAN, iterative 2 rounds); Stage 3 = expert interviews (QUAL + QUAN feasibility); Stage 4 = clinical evaluation (QUAL + QUAN). |
| 3. Description of each method: how was each method sampled, how were data collected and analysed | Section 2.2–2.7, 3 | Qualitative strands: (a) Think-aloud protocol (Stages 2 & 4) — convenience sampling via recruitment posters; university/community settings (Stage 2) and outpatient settings (Stage 4); sessions audio/screen-recorded and field-noted; inductive content analysis by two independent coders (LW + MZ), guided by Nielsen's 10 Usability Heuristics. (b) Semi-structured expert interviews (Stage 3) — purposive sampling of n = 18 HCPs from two medical centers; private rooms; 20–40 min; CFIR framework for deductive coding; two-coder consensus. Quantitative strand: SUS and NASA-TLX administered after each session (Stages 2 & 4); one-way ANOVA with Welch's correction and Games-Howell post hoc tests; SPSS v29. Demographics and clinical characteristics summarised descriptively (Tables 1, 3, 5). |
| 4. Description of where integration has occurred, how it has occurred and who has participated in it | Section 2.6, 4 | Integration occurred at the interpretation stage. After each iterative stage, qualitative themes (usability problems, implementation barriers) were triangulated with quantitative SUS/NASA-TLX scores to identify convergence or divergence, and findings were jointly interpreted by LW and MZ (Section 2.6, 4.1–4.2). The PDA was iteratively redesigned between rounds based on the integrated findings. Stage-level integration: Stage 2 qualitative issues informed prioritised redesign decisions validated by improved SUS scores in Round 2; Stage 3 CFIR-based barriers guided clinical workflow adaptation; Stage 4 integration confirmed final usability and patient-perceived utility. Integration was conducted by the core research team (LW and MZ) during regular research meetings. |
| 5. Description of the limitations of one method and how these were overcome using the other method | Section 5 | Limitations acknowledged in Section 5: (a) Qualitative think-aloud data are subject to observer and social desirability effects; quantitative SUS benchmarks provide an objective, validated reference point to cross-validate subjective reports. (b) SUS/NASA-TLX are self-reported scales sensitive to individual variability; qualitative data identify specific interaction problems that explain score variance. (c) Convenience sampling in Stages 2 and 4 limits representativeness; Stage 3 purposive expert sample provides depth on real-world clinical implementation. (d) Member checking was not conducted (data de-identified); dual-coder consensus and quantitative triangulation serve as alternative validity checks. |
| 6. Description of any insights gained from mixing or integrating methods — what was found that would not have been found using only one of the methods | Section 4.1–4.2, 6 | The Discussion (Section 4.1–4.2) and Conclusions (Section 6) describe insights uniquely enabled by mixing methods: (a) SUS scores first reached 'Good' usability from the expert interview stage onward (Stage 3: mean = 77.83; Stage 4: mean = 73.65), yet qualitative analysis continued to identify residual issues with icon comprehension and information density that quantitative scores alone would have obscured. (b) Expert interviews revealed CFIR-level adoption barriers (e.g., workflow disruption, cost and formulary concerns) that would not appear in patient-facing usability metrics. (c) Integration confirmed that design changes were associated with improvements in SUS scores across stages, validating the HCD iteration process. (d) Convergence of patient qualitative themes with NASA-TLX workload reductions suggesting reduced cognitive workload may support informed decision-making. |

*Abbreviations: CFIR = Consolidated Framework for Implementation Research; HCD = human-centered design; HCP = healthcare professional; NASA-TLX = National Aeronautics and Space Administration Task Load Index; PDA = patient decision aid; QUAL = qualitative; QUAN = quantitative; SUS = System Usability Scale.*
