# Supplementary material for: Digital Patient Decision Aid for Antiobesity Medications: Mixed Methods Study of Human-Centered Design and Usability Evaluation
Source: J Med Internet Res. 2026 May 15;28:e89428. doi: 10.2196/89428 (PMC13221622; doi:10.2196/89428)
Supplement: Multimedia Appendix 2 [file jmir_v28i1e89428_app2.docx]

# Consolidated Criteria for Reporting Qualitative Research (COREQ) Checklist

*Reference: Tong A, Sainsbury P, Craig J. Consolidated criteria for reporting qualitative research (COREQ): a 32-item checklist for interviews and focus groups. Int J Qual Health Care. 2007;19(6):349–357.*

Scope: Applies to qualitative components across Stages 2 (think-aloud, n = 78), Stage 3 (expert interviews, n = 18), and Stage 4 (patient interviews, n = 78).

| **No.** | **Guide Question / Description** | **Reported** | **Section / Page** |
| --- | --- | --- | --- |
| **Domain 1: Research Team and Reflexivity** | | | |
| **Personal characteristics** | | | |
| 1 | Interviewer/facilitator: Which author(s) conducted the interview or focus group? | Yes | Methods Section 2.3, Section 2.4, Section 2.5 — Think-aloud sessions and expert interviews conducted by the first author (LW) under supervision of the corresponding author (MZ). |
| 2 | Credentials: What were the researcher's credentials, e.g. PhD, MD? | Partial | Author byline — LW: graduate researcher; MZ: corresponding/senior researcher. Specific academic degrees not explicitly stated in text. |
| 3 | Occupation: What was their occupation at the time of the study? | Partial | Affiliations listed — researchers at academic institution; occupation not separately stated in Methods. |
| 4 | Gender: Was the researcher male or female? | No | Gender of interviewers not reported. |
| 5 | Experience and training: What experience or training did the researcher have? | Partial | Methods Section 2.3, 2.4 — Researchers trained in HCD methodology, usability testing, and qualitative content analysis; prior publications in PDA interface design cited (refs [46], [56], [57]). |
| **Relationship with participants** | | | |
| 6 | Relationship established: Was a relationship established prior to study commencement? | No | Not reported. No prior relationship described between researchers and participants. |
| 7 | Participant knowledge of the interviewer: What did the participants know about the researcher? | No | Not explicitly reported in the manuscript. |
| 8 | Interviewer characteristics: What characteristics were reported about the interviewer/facilitator? e.g. Bias, assumptions, reasons and interests in the research topic. | Yes | Methods Section 2.3–2.6 — The research team acknowledged prior experience in PDA design and usability research and minimized potential bias through independent coding and consensus discussion. |
| **Domain 2: Study Design** | | | |
| **Theoretical framework** | | | |
| 9 | Methodological orientation and theory: What methodological orientation was stated to underpin the study? e.g. grounded theory, discourse analysis, ethnography, phenomenology, content analysis. | Yes | Methods Section 2.3, 2.4, 2.5 — Inductive content analysis (Elo & Kyngäs [ref 45]); usability evaluation guided by Nielsen's 10 Usability Heuristics [ref 63]; expert interviews framed by Consolidated Framework for Implementation Research (CFIR) [refs 47, 48]. |
| **Participant selection** | | | |
| 10 | Sampling: How were participants selected? e.g. purposive, convenience, consecutive, snowball. | Yes | Methods Section 2.3 — Stage 2 (think-aloud): convenience sampling via recruitment posters at university and community settings; Section 2.4 — Stage 3 (expert interviews): purposive sampling of healthcare professionals (HCPs) at two medical centers; Section 2.5 — Stage 4 (clinical evaluation): convenience sampling of outpatients. |
| 11 | Method of approach: How were participants approached? e.g. face-to-face, telephone, mail, email. | Partial | Methods Section 2.3 — Recruited via posted flyers (university/community); Section 2.4 — HCPs recruited at two medical centers (face-to-face approach implied); method of initial contact not explicitly described. |
| 12 | Sample size: How many participants were in the study? | Yes | Methods Section 2.3, Table 1 — Stage 2: n = 78 (Round 1: n = 38; Round 2: n = 40); Section 2.4 — Stage 3: n = 18 HCPs; Section 2.5 — Stage 4: n = 78 patients. |
| 13 | Non-participation: How many people refused to participate or dropped out? Reasons? | Yes | Methods Section 2.3–2.5 — No participant withdrawals were observed; non-participation rates were minimal and did not affect the overall sample. |
| **Setting** | | | |
| 14 | Setting of data collection: Where was the data collected? e.g. home, clinic, workplace. | Yes | Methods Section 2.3 — Stage 2: university laboratory and community venues; Section 2.4 — Stage 3: two medical centers, conducted in private rooms to ensure confidentiality; Section 2.5 — Stage 4: outpatient clinic settings. |
| 15 | Presence of non-participants: Was anyone else present besides the participants and researchers? | No | Not reported. Private rooms used in Stage 3; presence of others not described for Stages 2 and 4. |
| 16 | Description of sample: What are the important characteristics of the sample? e.g. demographic data, date. | Yes | Results Section 3.1, Table 1 —Stage 2 participants: age, sex, and education level described.; Table 3 — Stage 3 HCPs: specialty, years of practice; Table 5 — Stage 4 patients: age, gender, BMI, comorbidities. Study period: August 2022–November 2025. |
| **Data collection** | | | |
| 17 | Interview guide: Were questions, prompts, guides provided by the authors? Was it pilot tested? | Yes | Methods Section 2.3, 2.4 — Interview guides were developed based on established frameworks and iteratively refined during early data collection. |
| 18 | Repeat interviews: Were repeat interviews carried out? If yes, how many? | Yes | Methods — No repeat interviews conducted. Stage 2 employed two iterative design rounds (Round 1 and Round 2) with different participant cohorts, not the same individuals. |
| 19 | Audio/visual recording: Did the research use audio or visual recording to collect the data? | Yes | Methods Section 2.3 — Think-aloud sessions were audio/screen-recorded; Section 2.4, Section 2.5 — Expert and patient interviews recorded for transcription. |
| 20 | Field notes: Were field notes made during and/or after the interview or focus group? | Yes | Methods Section 2.3 — Field observations and notes were documented during think-aloud sessions to capture interaction behaviors. |
| 21 | Duration: What was the duration of the interviews or focus group? | Partial | Methods Section 2.4 — Expert interviews: approximately 20–40 minutes; Stage 2 think-aloud sessions duration not explicitly stated (implied by task-based protocol). |
| 22 | Data saturation: Was data saturation discussed? | Yes | Methods Section 2.3–2.5 — Data saturation was not formally assessed, as the study followed an iterative usability-focused human-centered design approach with predefined sample sizes. |
| 23 | Transcripts returned: Were transcripts returned to participants for comment and/or correction? | No | Limitations Section 5 — Member checking not conducted; data were de-identified to protect participant confidentiality, precluding transcript return. |
| **Domain 3: Analysis and Findings** | | | |
| **Data analysis** | | | |
| 24 | Number of data coders: How many data coders coded the data? | Yes | Methods Section 2.3, Section 2.4, Section 2.5 — Two independent coders (LW and MZ) performed qualitative content analysis; discrepancies resolved through consensus discussion. |
| 25 | Description of the coding tree: Did authors provide a description of the coding tree? | Partial | Results Section 3.2, Section 3.4 — Themes and categories reported in tables and text; full hierarchical coding tree not presented. Stage 3 themes organized by CFIR domains. |
| 26 | Derivation of themes: Were themes identified in advance or derived from the data? | Yes | Methods Section 2.3, 2.5 — Themes derived inductively from data guided by Nielsen's Heuristics; Section 2.4 — Stage 3 themes deductively mapped to CFIR framework domains. |
| 27 | Software: What software, if applicable, was used to manage the data? | Yes | Methods Section 2.3–2.6 — Qualitative data were coded manually without dedicated qualitative software; SPSS v29 was used for quantitative analysis. |
| 28 | Participant checking: Did participants provide feedback on the findings? | No | Limitations Section 5 — Participant checking (member checking) was not conducted due to data de-identification. |
| **Reporting** | | | |
| 29 | Quotations presented: Were participant quotations presented to illustrate the themes/findings? Was each quotation identified? | Yes | Results Section 3.2, Section 3.4 — Representative participant quotations included to illustrate usability themes and implementation barriers; quotations labeled by participant type. |
| 30 | Data and findings consistent: Was there consistency between the data presented and the findings? | Yes | Results Section 3.2–3.5 — Qualitative themes substantiated by participant quotations and corroborated with quantitative SUS/NASA-TLX scores; integrated interpretation presented in Section 4. |
| 31 | Clarity of major themes: Were major themes clearly presented in the findings? | Yes | Results Section 3.2, Section 3.4 — Major themes clearly organized and labeled; usability categories (Stages 2 and 4) and implementation domains (Stage 3) tabulated. |
| 32 | Clarity of minor themes: Is there a description of diverse cases or discussion of minor themes? | Partial | Results Section 3.2, Section 3.4 — Predominant themes reported; minority viewpoints or negative cases not explicitly discussed as such. |
